# Comparative Population Dynamics of *Schizothorax wangchiachii* (Cyprinidae: Schizothoracinae) in the Middle Reaches of the Yalong River and the Upper Reaches of the Jinsha River, China

**DOI:** 10.3390/ani13132209

**Published:** 2023-07-05

**Authors:** Zhi He, Kuo Gao, Hongjun Chen, Deying Yang, Yong Pu, Li Zheng, Yuanyuan Jiao, Jinxin Xiong, Qiqi Chen, Bolin Lai, Mingwang Zhang, Ziting Tang, Taiming Yan

**Affiliations:** College of Animal Science and Technology, Sichuan Agricultural University, Chengdu 611130, China; zhihe@sicau.edu.cn (Z.H.); gk2238596556@163.com (K.G.); jet4rhzd1@163.com (H.C.); deyingyang@sicau.edu.cn (D.Y.); 18483284342@163.com (Y.P.); zli313467@163.com (L.Z.); 17381978871@163.com (Y.J.); xiongjinxin2013@163.com (J.X.); cqq17740194917@126.com (Q.C.); bolin983152336@126.com (B.L.); 13816@sicau.edu.cn (M.Z.); 15308110569@163.com (Z.T.)

**Keywords:** the upper reaches of the Yangtze River, per-recruit analysis, age, growth, growth characteristics, fisheries stock management

## Abstract

**Simple Summary:**

Ecological characteristics of the two economically important *Schizothorax wangchiachii* populations from the upper Yangtze River were compared. Their age structures were similar, but the growth coefficient, initial sexual maturity age and age at first capture were significantly different between the two populations. Both populations were in an overfishing state due to the current fishing intensity. These results can provide information for planning additional resource protection for *Schizothorax wangchiachii*.

**Abstract:**

To explore the differences in the growth characteristics and population dynamics of *Schizothorax wangchiachii* populations in the Jinsha River (JSR) and the Yalong River (YLR), samples were collected in the upper reaches of the JSR (*n* = 230) from 2019 to 2020 and the middle reaches of the YLR (*n* = 187) from 2017 to 2018. In the JSR and YLR populations, the age range was 11 and 12 years old, respectively, and the best growth equation was the Von Bertalanffy equation. The comparative analysis of the two populations showed that the growth coefficient, initial sexual maturity age and age at first capture of the YLR population were greater than those of the JSR population. Comparing the mortality rates of the two groups, we found that the YLR population had the higher female mortality rate (0.658 years^−1^) and the lower male mortality rate (0.453 years^−1^). Our assessment of the three natural mortality rates showed that the *F_cur_* of both male and female populations was greater than *F*_25%_, indicating that both populations were in an overexploited state. Therefore, we suggest considering the two groups as separate protection units and implementing management measures such as ecological regulation, restoration of tributary habitat and strengthening of fishing ban monitoring to protect their resources.

## 1. Introduction

Determining the age of fish is the basis for fish population dynamic assessments. Based on age data and a population dynamic assessment, parameters such as fish growth parameters, yield per recruit and exploitation rate can be calculated [1]; the results could clarify the development status of the population and the trends in resource decline, providing a basis for formulating relevant protection measures [2,3,4]. Studies has shown that the growth characteristics of fish were not uniform, and there were spatial and temporal differences [5]. However, traditional fishery resource assessments are usually based on fixed growth parameters. This might lead to inappropriate decisions regarding fishery management and increase the risk of fishery management [5,6]. Therefore, understanding the specific age structure and growth parameters of different populations is important for improving fishery resource management [7].

*Schizothorax wangchiachii* is an endemic fish in the upper reaches of the Yangtze River and is mainly distributed in the JSR and YLR basins [8,9]. When used as a food source, the fish is fresh and delicious, so *S. wangchiachii* has become a major catch in the JSR basin [8,9,10]. A resource survey conducted from 2004 to 2011 showed that the development and construction of cascade hydropower projects in the JSR and YLR led to rapid changes in the ecological environment of the waters in the region. As a result, the catch of *S. wangchiachii* was miniaturized and younger, and the natural population resources exhibited a downward trend [10,11]. Thus, conserving the resources of its existing population is essential. However, previous studies of *S. wangchiachii* have mainly focused on artificial reproduction, embryonic development, and genetic diversity [12,13,14], but there are no reports on its growth characteristics and population dynamics. At the same time, the growth characteristics of different fish populations vary, governed by different population life history parameters [5,15]. Therefore, understanding the population dynamics of different populations would be helpful for developing protective measures [3].

In this study, *S. wangchiachii* were collected in the JSR and YLR basins from 2017 to 2020 to collect age data and use them to determine the age structure and growth characteristics of the two populations. The per-recruit model was used to evaluate the population dynamics and understand the current resource status. We provide a scientific basis for the management and rational utilization of the resources of *S. wangchiachii* by exploring the following: (1) the differences in the growth parameters of different populations of *S. wangchiachii*, (2) the state of resource exploitation of different groups, and (3) the optimal fishing standards.

## 2. Materials and Methods

### 2.1. Sample Collection

The JSR and the YLR (the largest branch of the Jinsha River) are near the Qinghai-Tibet Plateau and are both typical mountain rivers [16,17]. The spatial distribution of multi-year average precipitation in the upper reaches of the JSR is in the range of 30 to 1000 mm, and the water vapor content over the basin is less than 15 mm, which is relatively scarce [18,19]. The lowest annual average water temperature in the upper reaches of the JSR is 0.3 °C, with a short ice cover period. However, the middle reaches of the YLR have a long winter ice cover period, with a water temperature of 0 °C [19,20]. The average annual flow of the Shigu section is approximately 2300 m^3^/s [21].

From April 2017 to May 2018, in the middle reaches of the YLR and its tributary Xianshui River, and from September 2019 to October 2019 and from June 2020 to July 2020, in the upper reaches of the JSR (Benda-Shigu), *S. wangchiachii* was collected by means of purchase from fishermen and self-fishing using gill nets (2–5 cm mesh), ground cage fishing nets (length, width and height: 15 m, 0.3 m and 0.3 m, respectively), as well as via identification in the ichthyofaunal monographs in China [22,23]. Random sampling was performed within the sampling section, gill nets and ground cage fishing nets were placed at 8 am every day and catches were collected at 8 am the next day.

**Figure 1:**
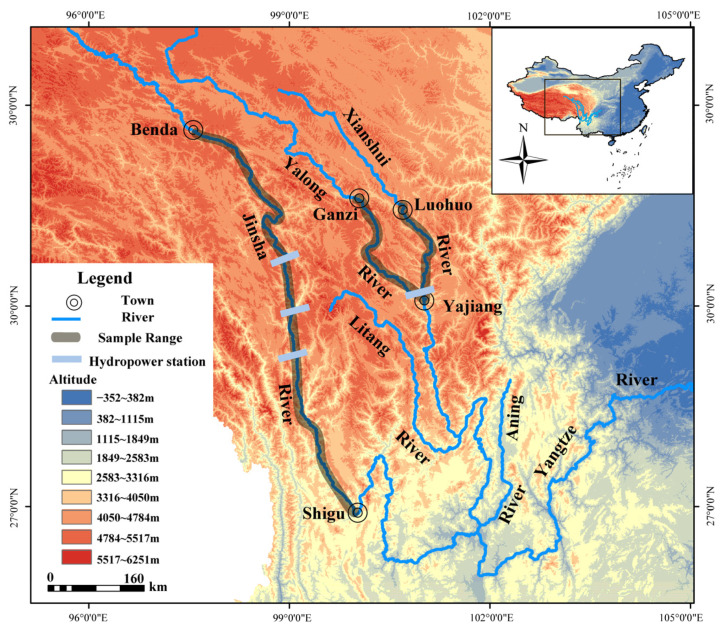
Sampling range of *Schizothorax wangchiachii*.

The fish were euthanized with MS-222 (98%), and then the body length (BL, accurate to 0.1 cm), body weight (BW, accurate to 0.1 g) and other basic biological data were measured and recorded. On-site dissection was conducted to determine the sex and gonad stage, and the lapillus was extracted, stored in a 1.5 mL centrifuge tube filled with ethanol (95%), and brought back to the laboratory for treatment (Figure 1).

### 2.2. Sample Preparation

Neutral resin was used to fix the concave surface of the microotolith on the slide. After natural air drying, 800 # to 3000 # abrasive paper and water were used to polish it, and the slide was observed under the microscope until the growth center of the otolith could be clearly observed. Then, the otolith grinding plate was washed with water. It was polished with polishing paper until the wheel pattern was completely clear. Finally, the resin was dissolved, turned over, dried, and fixed again, and the other side of the otolith was polished using the same method. Once the wheel pattern was clear, observations were made and photographs were taken with the Leica Dc500 digital photography system, and the pictures were saved. In the identification of annual rings, the dividing line between the dense belts formed in autumn and winter and the sparse belts formed in spring and summer of the following year were used to determine the age mark.

### 2.3. Age Assessment and Verification

Age was calculated as follows. When the number of annual rings on the otolith was *n*, *n* was recorded as the age. If a new ring outside the nth ring had not yet fully formed but a few dense bands appeared, the age was recorded as (*n* + 1) [24]. Age was determined without knowledge of the basic biological information of *S. wangchiachii*. Two annual ring counts were carried out; if the results were different, counting was performed a third time. Age estimates were compared by calculating the index of average percentage error (*IAPE*) between researchers and between pairs of aging materials [25,26]. To calculate the *IAPE*, the formula of Beamish and Fournier was used as follows:

(1) $${IAPE}_{j}=100\%\times \frac{1}{R}\sum_{i=1}^{R}\frac{\left|x_{ij}-x_{j}\right|}{x_{j}}$$

where *x_ij_* is the *i*th age determination of the *j*th fish, *x_j_* is the average age calculated for the *j*th fish, and *R* is the number of times the age of each fish was determined.

### 2.4. Modeling the Length–Weight Relationship and Growth

The power function $W=aL^{b}$ was used to fit the relationship between body weight and body length of *S. wangchiachii* [27]. Analysis of covariance (ANOVA) was used to analyze the difference in the body length–weight relationship between male and female individuals [28]. A *t* test was used to compare the allometric growth index (b) and 3 to determine whether *S. wangchiachii* was growing at a uniform speed [27,29].

Three equations were used to describe the relationship between fish age and growth, including the von Bertalanffy equation (VBGF), logistic equation (LGF) and Gompertz equation (GGF). The minimum residual sum of squares (RRS) and Akaike information criterion (AIC) were used to determine the optimal growth equation, for which the fitting degree was determined using the determination coefficient R^2^. The formulas are as follows [30,31,32]:

(2) $$\mathrm{VBGF}:\;L_{t}=L_{\infty }\left(1-e^{-k(t-t_{0}}\right);$$

(3) $$\mathrm{GGF}:\;L_{t}=\frac{L_{\infty }}{{1+e}^{-k(t-t_{0})}};$$

(4) $${\mathrm{LGF}:\;L_{t}=L}_{\infty }e^{-e-k(t-t_{0})}$$

where *L_t_* is the expected total length at age *t* years, *L_∞_* is the asymptotic total length, *k* is the growth parameter indicating the rate at which *S. wangchiachii* grows toward *L_∞_*, and *t*_0_ is the theoretical age at zero total length.

The *AIC* was used to determine the optimal growth model. The formula is as follows [33]:

(5) $$AIC=-2LnL\left(p1,\ldots ,\;pm,{\;\sigma }^{2}\right)+2\;m$$

where *m* is the model parameter; *LnL* (*p*1,…, *pm*, σ^2^) is the residual sum of squares; and the optimal model is the one with the minimum AIC value.

### 2.5. The Ages at First Sexual Maturity

The body length was divided into different sections with a group distance of 20 mm, and then the sexual maturity (gonad development reached stage Ⅲ to Ⅵ) ratio of female and male populations in the section was counted. Then, the median body length group distance and sexual maturity ratio of each section were fitted using logistic regression, and the formula is as follows [34]:

(6) $$P=1/\left[1+e^{-k\left(L_{mid}-L_{50}\right)}\right]$$

where *P* is the sexual maturity ratio of each body length section; *L_mid_* is the median body length group spacing (20 mm); *L*_50_ is the body length of the first sexual maturity; and *k* is the slope.

The proportion of sexually mature individuals in each age group was counted by sex, and logistic regression was performed with each age group. The formula is as follows [35]:

(7) $$G=1/\left[1+e^{-k\left(T-T_{50}\right)}\right]$$

where *G*: sexual maturity ratio of each age group; *T*: age; *T*_50_: age of first sexual maturity; and *k*: slope.

### 2.6. Estimation of Mortality

The Chapman–Robson (CR) method was used to estimate the total mortality rate (*Z*) of *S. wangchiachii*, and the formula is as follows [36]:

(8) $$Z=ln\frac{1+\bar{T}-t_{c}-\frac{1}{N}}{\bar{T}-t_{c}}-\frac{\left(N-1\right)\left(N-2\right)}{N\left[N\left(\bar{T}-t_{c}\right)+1\right]\left[N+N\left(\bar{T}-t_{c}\right)-1\right]}$$

where *t_c_* is the starting age; $\bar{T}$ is the average age of the sample when it is greater than *t_c_*; and *N* is the number of samples when it is greater than *t_c_*.

The following two empirical equations were applied to estimate the natural mortality rate (*M*) [37]:

(9) $$M_{T}=4.899\;t_{max}^{-0.916}$$

(10) $$M_{G}=4.118\;k^{0.73}L_{\infty }^{-0.33}$$

(11) $$M_{R}=\frac{M_{G}+M_{G}}{2}$$

where the parameters are as follows: *M_T_*, natural mortality calculated according to growth parameters; *M_G_*, natural mortality estimated by the limit age method; *k*, the growth parameter; *M_R_*, the median of the *M_G_* and *M_T_* was selected as the reference natural mortality; *t*_0_, the age at zero length; and $L_{\infty }$, the asymptotic length. In addition, *t_max_* is the longevity of *S. wangchiachii,* which was estimated using the following formula [38]:

(12) $$t_{\max}=t_{0}+\frac{2.996}{k}$$

The fishing mortality rate (*F*) was calculated as *F = Z* − *M*. The estimates of *M* and *F* were assumed to be constant throughout the fish lifespan.

### 2.7. Per-Recruit Analysis

Yield per recruit (*YPR*) and spawning stock biomass per-recruit (*SSB*/*R*) models were developed for the pooled data. The *YPR* and *SSB*/*R* were calculated using the following formulas [39]:

(13) $$YP𝑅=\frac{Y}{R}\sum_{t=t_{r}}^{t_{max}}\frac{FS_{t}}{FS_{t}+M}e^{\left(-FS_{t}-M\right)\left(t-t_{c}\right)}e^{-M\left(t_{c}-t_{r}\right)}\left(1-e^{-FS_{t}-M}\right)\;aL_{t}^{b}$$

(14) $$SPR=\frac{SSBR_{F}}{SSBR_{F=0}}$$

(15) $$𝑆𝑆𝐵𝑅=\frac{SSB}{R}\sum_{t=t_{r}}^{t_{max}}e^{\left(-FS_{t}-M\right)\left(t-t_{c}\right)}e^{-M\left(t_{c}-t_{r}\right)}aL_{t}^{b}G_{t}$$

where *Y* is the total catch; *R* is the total supplementary population; *SSB* is the total number of parent fish in the breeding population; *SPR* is the spawning potential ratio, defined as the value of dividing the unit supplementary amount of parental quantity by the unit supplementary amount of parental quantity without the fishing mortality rate under a certain fishing mortality coefficient; *SSBR* is the unit supplementary amount of parent fish; *F* is the fishing mortality rate; *M* is the natural mortality rate; *t_max_* is the maximum observed age; *t_r_* is the age at recruitment, which was the youngest age in the catch; *t_c_* is the age at first capture (*t_max_*, *t_r_* and *t_c_* are obtained from age frequency distribution analysis); *k* is the growth parameter; *t*_0_ is the hypothetical age at zero length; $L_{\infty }$ is the asymptotic length in the von Bertalanffy growth function; *a* and *b* are parameters in the weight–length relationship; *G_t_* is the proportion of mature fish at age *t* (*S. wangchiachii* spawns just once each year), which was modeled by a previously published logistic function [40]; and *S_t_* is the gear selectivity coefficient for fish of age *t* and was set to ‘knife edge’ selectivity as follows:

*S_t_* = 0(*t* < *t_c_*) or 1 *(t* ≥ *t_c_*) (16)

### 2.8. Biological Reference Points

To determine the fishery status of the *S. wangchiachii* stock downstream of the JSR, the current fishing mortality (*F_cur_*) was compared with four *F*-based *BRPs*: *F_max_*, where the fishing mortality rate produces the maximum *YPR*; *F*_0.1_, which refers to fishing mortality where the slope of the *YPR* curve was 10% of the slope at the origin [41]; and *F*_25%_ and *F*_40%_, which were fishing mortality rates at which the *SPR* was 25% and 40%, respectively [42].

### 2.9. Statistical Analysis

Data are expressed as the mean ± standard error. SPSS 20.0, GraphPad Prism 8.0 and Excel 2018 were used to process and analyze the data. When *p* < 0.05, the difference was considered significant. Images in this paper were processed with CorelDRAW 2019, Adobe Illustrator 2023 and ArcGIS10.4.1.

## 3. Results

### 3.1. Sample Characteristics

The *BL* of *S. wangchiachii* in YLR and JSR ranged from 101.0 to 401.0 mm and 88.0 to 334.0 mm, and the *BW* ranged from 20.6 to 1011.2 g and 10.6 to 640.3 g, respectively (Table 1). The mean weights of YLR and JSR *S. wangchiachii* were 232.6 ± 167.3 and 131.6 ± 99.15 g, respectively. The average body length and body weight of the YLR population were significantly greater than those of the JSR population *(p* < 0.05).

### 3.2. Age Structure and Verification

**Figure 2:**
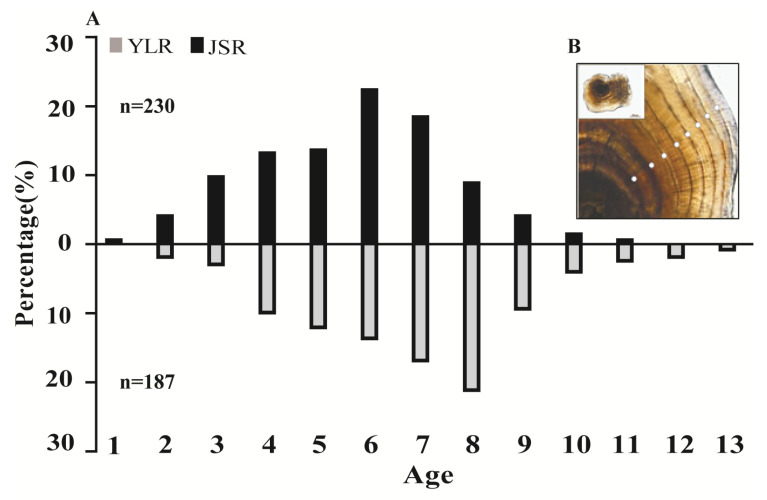
Age composition and annulus characteristics of the lapillus in *Schizothorax wangchiachii*. YLR, Yalong River; JSR, Jinsha River. (**A**) The age composition of the Jinsha River and Yalong River populations; (**B**) the annual characteristics of otoliths, and each white dot represents one growth cycle, per year.

There was an approximately circular nucleus in the center of the otolith, and the transparent and opaque zones on the otolith were arranged in an irregular concentric circle. The transparent band was narrow and transparent, while the dark band was opaque, allowing for clear growth blockages. The junction of the bright and dark bands was the annulus. The ring spacing on the otolith shows a regular decreasing trend, with larger ring spacing near the central nucleus, while ring spacing away from the central nucleus gradually decreases (Figure 2B).

We considered the dividing line between the dense zone formed in autumn and winter and the sparse zone formed in spring and summer of the following year to be the annual ring. In transmitted light, the annual rings appeared as dark bands, so counting the dark bands as ages and calculating the average percentage error three times ensured the accuracy of identification. In the YLR and JSR groups, the reliability of the age estimates had a low *IAPE* (1.51% and 1.68%), indicating high accuracy. There were certain differences in the age composition of the two populations. The age range of the population in the YLR was 2 to 13 years, and the dominant age group was 5 to 9 years, representing 65.77% of the total. The age range of the population in the JSR was 1 to 11 years, and the dominant age group was 4 to 7 years, representing 77.39% of the total (Figure 2A).

### 3.3. Growth Modeling

The analysis of covariance (ANOVA) showed that there was no significant difference in the relationship between body length and body weight of the fitted male and female individuals in the YLR population (*F* = 0.109, *P* = 0.741, *p >* 0.05). This indicated that regression analysis of the relationship between body length and body weight could be carried out for male and female individuals combined. The same was true for the JSR population (*F* = 0.035, *P* = 0.852, *p >* 0.05). There was no significant difference between the *b* value and 3 (YLR: t = 1.2177 ≤ t_0.01_ = 2.602; JSR: t = 2.4230 ≤ t_0.01_ = 2.598), indicating that the growth of the fish in the YLR and JSR populations was uniform (Table 2).

Compared with other models, the growth model based on the von Bertalanffy equation had a lower AIC value (YLR 66.49/JSR 80.10) (Table 2), and the results of the χ^2^ test indicated that the difference between the theoretical value and the actual value was not significant for the von Bertalanffy equation (*p* > 0.05). These results suggested that the von Bertalanffy equation can better reflect the growth of *S. wangchiachii*.

### 3.4. Growth Parameters

Table 3 and Table 4 show the biological parameters of the two populations of *S. wangchiachii*. In the YLR population, the ages at first sexual maturity of female and male *S. wangchiachii* were 7.09 and 5.41 years, respectively, and the ages at first capture (*t_c_*) were 6.82 and 7.10 years, respectively. The total mortality (*Z*) of females and males was 0.658 and 0.453 year^−1^, respectively. According to the growth parameters, the natural mortality (*M_G_*) of female and male fish was 0.06 and 0.07 year^−1^, respectively, and the corresponding fishing mortality (*F_cur_*) was 0.59 and 0.38 year^−1^, respectively. The natural mortality (*M_T_*) of female and male fish estimated by the limit age method was 0.13 and 0.15 year^−1^, and the corresponding *F_cur_* was 0.52 and 0.30 year^−1^, respectively. The median of the *M_G_* and *M_T_* was selected as the reference natural mortality (*M_R_*), the *M_R_* of female and male fish were 0.09 and 0.11 year^−1^, and the corresponding *F_cur_* was 0.56 and 0.34 year^−1^, respectively.

In the JSR population, the ages at first sexual maturity of female and male *S. wangchiachii* were 6.09 and 5.19 years, and the *t_c_* values were 5.49 and 5.36 years, respectively. The *Z* values of females and males were 0.504 and 0.532 year^−1^, respectively. According to the growth parameters, the *M_G_* of female and male fish were 0.06 and 0.06 year^−1^, and the corresponding *F_cur_* values were 0.44 and 0.47 year^−1^, respectively. The *M_T_* of female and male fish obtained by the limit age method were 0.14 and 0.12 year^−1^, and the corresponding *F_cur_* values were 0.36 and 0.41 year^−1^, respectively. The *M_R_* values of female and male fish were 0.10 and 0.09 year^−1^, and the corresponding *F_cur_* values were 0.40 and 0.44 year^−1^, respectively.

The growth parameter *k*, the age at initial sexual maturity and the age at initial catch of the YLR population were greater than those of the JSR population. The total mortality rate of female individuals in the YLR population was the highest, and the total mortality rate of male individuals was the lowest. The male age at first capture of the YLR population was the largest and that of the JSR population was the smallest.

### 3.5. Per-Recruit Analysis

**Figure 3:**
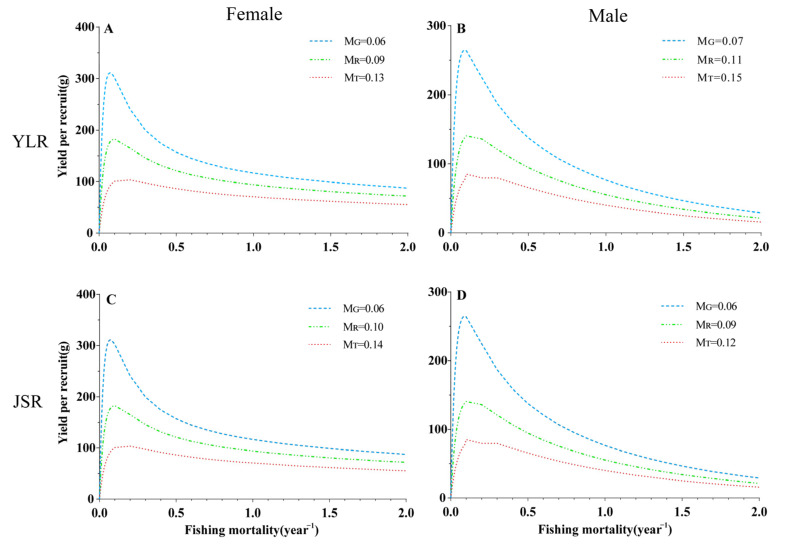
Yield per recruit (*YPR*) under different fishing mortality rates of *Schizothorax wangchiachii*. (**A**,**B**), the yield per-recruit curve under different fishing mortality rates of female and male *S. wangchiachii* in the middle reaches of the YLR; (**C**,**D**), the catch curve under different fishing mortality rates of male *S. wangchiachii* in the upper reaches of the JSR. YLR, Yalong River; JSR, Jinsha River; *M_T_*, natural mortality calculated according to growth parameters; *M_G_*, natural mortality estimated by the limit age method; *M_R_*, the reference natural mortality calculated from the medians of *M_T_* and *M_G_*.

**Figure 4:**
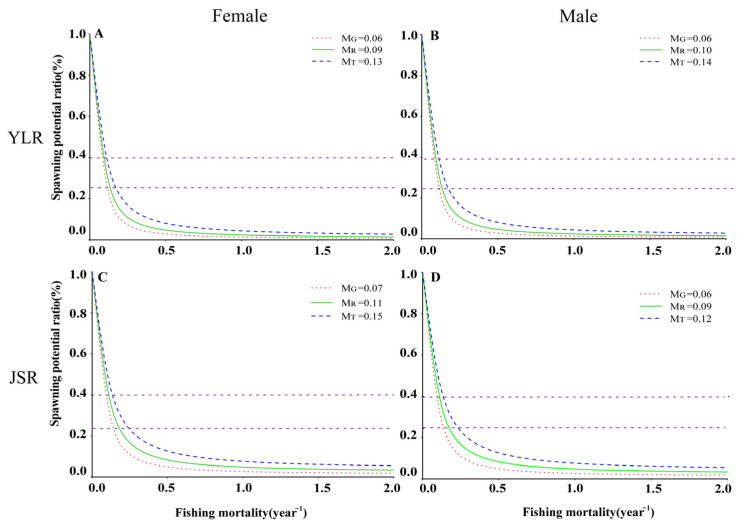
The spawning potential ratio (*SPR*) of *Schizothorax wangchiachii*. (**A**,**B**), the spawning potential ratio curve under different fishing mortality rates of female and male *S. wangchiachii* in the middle reaches of the YLR; (**C**,**D**), the spawning potential ratio curve under different fishing mortality rates of male *S. wangchiachii* in the upper reaches of the JSR. The dashed and dotted lines represent *SPR* at 25 and 40%, respectively. YLR, Yalong River; JSR, Jinsha River; *M_T_*, natural mortality determined according to growth parameters; *M_G_*, natural mortality estimated by the limit age method; *M_R_*, the reference natural mortality calculated from the medians of *M_T_* and *M_G_*.

Per-recruit analysis was conducted with the biological parameters. In the YLR population, the three natural mortality rates (*M_G_*, *M_R_*, *M_T_*) were used to fit the change trend of the spawning potential ratio (*SPR*) and yield per recruit (*YPR*) of female and male fish under different fishing intensities (Figure 3A,B and Figure 4A,B). The range of the spawning potential ratio (*SPR*) for females was 2.92 to 11.25%, while that for males was 5.91 to 18.57% (Table 4). Under the three natural mortality rates, the *F_cur_* of both female and male populations was greater than *F*_25%_, indicating that the population was in an overdeveloped state.

**Figure 5:**
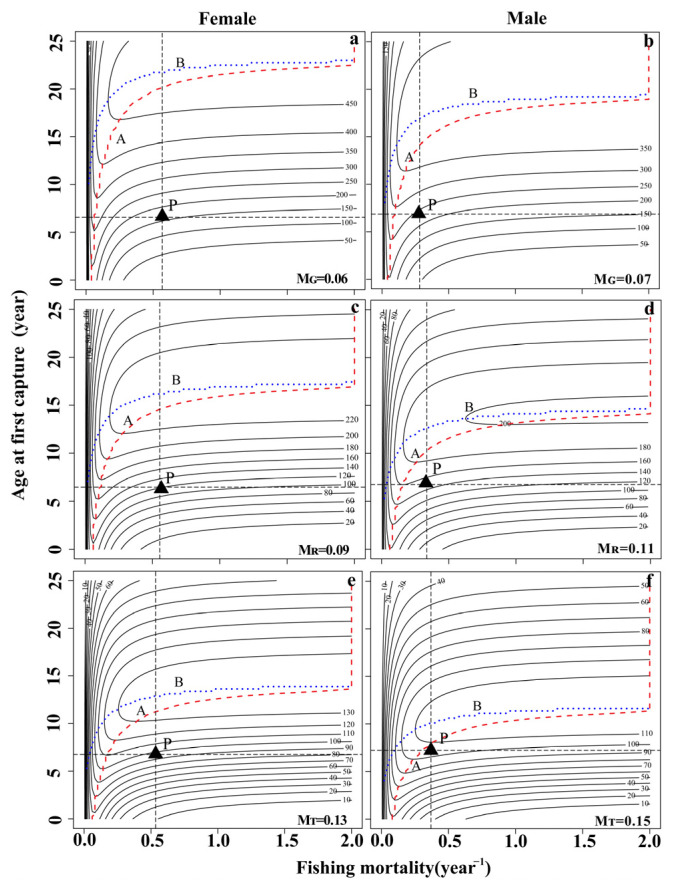
Isopleths describing the response of yield per recruit (*YPR*) to various fishing mortalities (*F*) and ages at first capture (*t_c_*) for *Schizothorax wangchiachii* in the YLR. (**a**,**c**,**e**) The response of *YPR* to *t_c_* and *F* under different natural mortalities in female *S. wangchiachii*; (**b**,**d**,**f**) the response of *YPR* to *t_c_* and *F* under different natural mortalities in male *S. wangchiachii*. The “P” point shows the current average fishing conditions. The optimal yield area in the figure is the area between Line A (optimal *t_c_* point line) and Line B (optimal *F* point line). *M_T_*, natural mortality was calculated according to growth parameters; *M_G_*, natural mortality was estimated by the limit age method; *M_R_*, the reference natural mortality was calculated from the medians of *M_T_* and *M_G_*.

**Figure 6:**
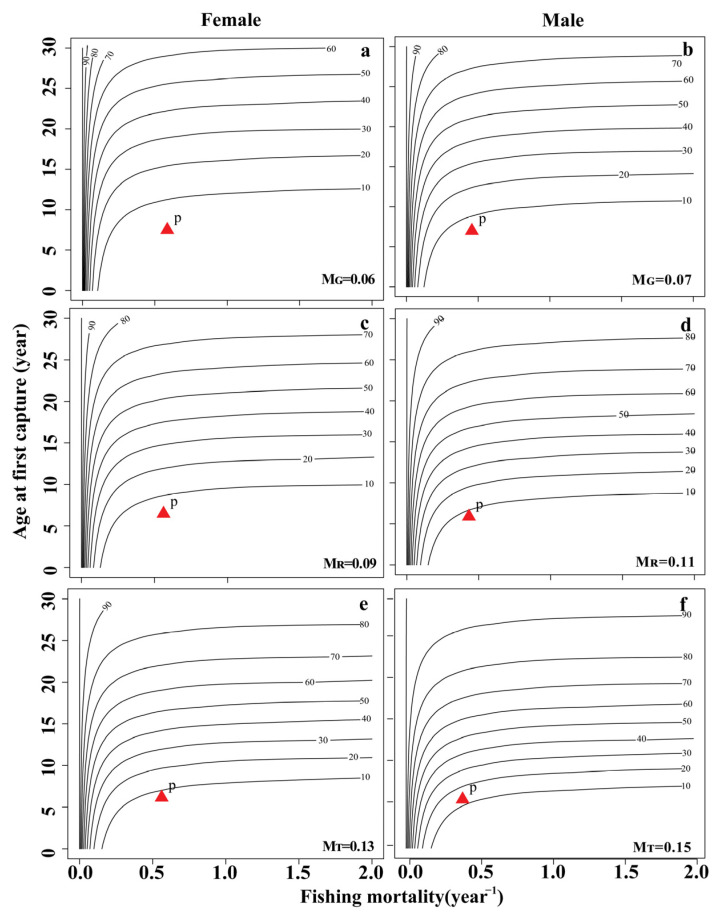
Isopleths of the spawning potential ratio (*SPR*) for *Schizothorax wangchiachii* in the middle reaches of the YLR. (**a**,**c**,**e**) The response of *SPR* to age at first capture (*t_c_*) and fishing mortality (*F*) under different natural mortalities in female *S. wangchiachii*; (**b**,**d**,**f**) the response of *SPR* to *t_c_* and *F* under different natural mortalities in male *S. wangchiachii*. The “P” point represents the current estimated *SPR*; *M_T_*, natural mortality was calculated according to growth parameters; *M_G_*, natural mortality was estimated by the limit age method; *M_R_*, the reference natural mortality was calculated from the medians of *M_T_* and *M_G_*.

Three natural mortality values were used to simulate the effects of different fishing intensities and starting ages on the *YPR* and *SPR* of female and male fish. The *YPR* value first increased rapidly and then decreased with increasing fishing intensity and maintained a relatively stable state (Figure 3A,B). Similarly, the *YPR* value also first increased and then decreased with increasing fishing age and natural mortality (Figure 5). The *SPR* value increased with increasing fishing age, decreased rapidly with increasing fishing intensity, and then remained stable (Figure 4A,B and Figure 6).

The catch per unit supplement curve (X axis: fishing death coefficient, Y axis: age at first capture (*t_c_*)) was drawn (Figure 5 and Figure 6). The optimal yield area in the figure was the area between Line A (optimal *t_c_* point connection) and Line B (optimal *F* point connection). In 2017–2018, the corresponding catch per unit supplementary number of females (*F* = 0.52–0.59, *t_c_* = 6.82 a) and males (*F* = 0.30–0.38, *t_c_* = 7.10 a) in the current fishery site did not fall within the range of the optimal yield area, indicating that the current population resources of *S. wangchiachii* in the middle reaches of the YLR are in an overdeveloped state (Figure 5 and Figure 6).

In the JSR populations, the range of the spawning potential ratio (*SPR*) for females was 2.38 to 7.59%, while that for males was 1.85 to 6.50% (Table 4). Based on the three natural mortality rates, the *F_cur_* of both female and male populations was greater than *F*_25%_, indicating that the population was in an overdeveloped state (Figure 3C,D and Figure 4C,D).

**Figure 7:**
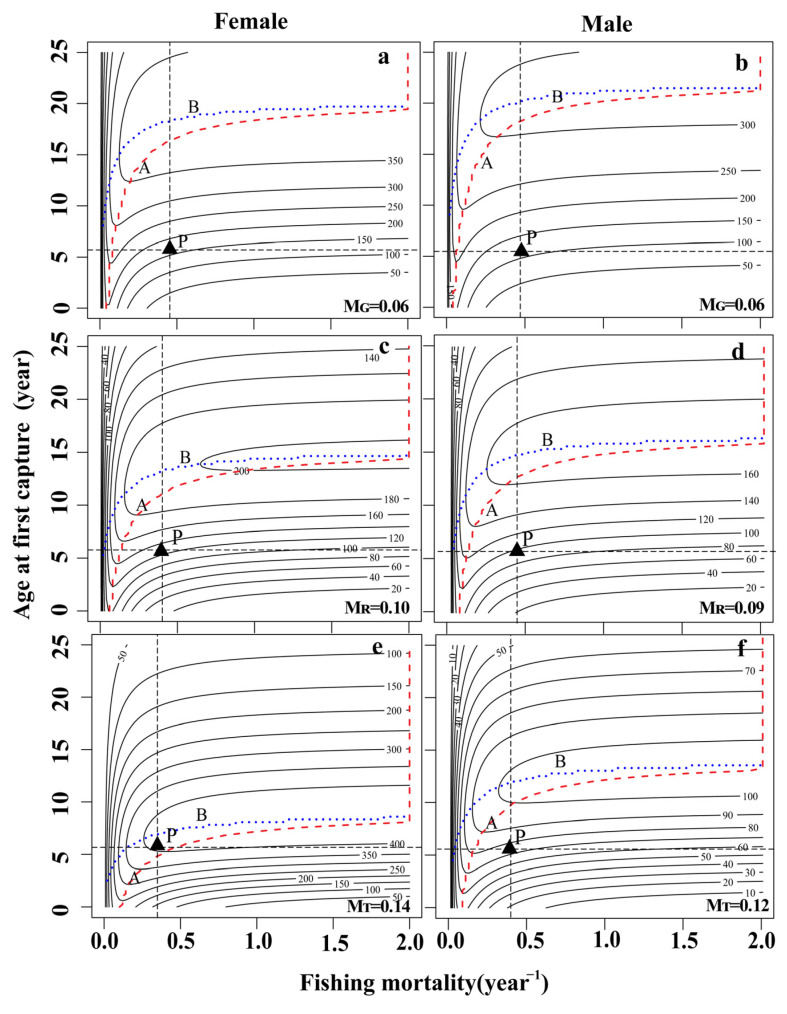
Isopleths describing the response of yield per recruit (*YPR*) to various fishing mortalities (*F*) and age at first capture (*t_c_*) for *Schizothorax wangchiachii* in the JSR. (**a**,**c**,**e**) The response of *YPR* to *t_c_* and *F* under different natural mortalities in female *S. wangchiachii*; (**b**,**d**,**f**) the response of *YPR* to *t_c_* and *F* under different natural mortalities in male *S. wangchiachii*. The “P” point is the current average fishing conditions. The optimal yield area in the figure is the area between Line A (optimal *t_c_* point line) and Line B (optimal *F* point line). *M_T_*, natural mortality was calculated according to growth parameters; *M_G_*, natural mortality was estimated by the limit age method; *M_R_*, the reference natural mortality was calculated from the medians of *M_T_* and *M_G_*.

Three natural mortality values were used to simulate the effects of different fishing intensities and starting ages on the *YPR* and *SPR* of female and male fish. The *YPR* value first increased rapidly and then decreased with increasing fishing intensity and maintained a relatively stable state (Figure 3C,D). Similarly, the *YPR* value first increased and then decreased with increasing fishing age and natural mortality (Figure 7). The *SPR* value increased with increasing fishing age, decreased rapidly with increasing fishing intensity, and then remained stable (Figure 4C,D and Figure 6).

**Figure 8:**
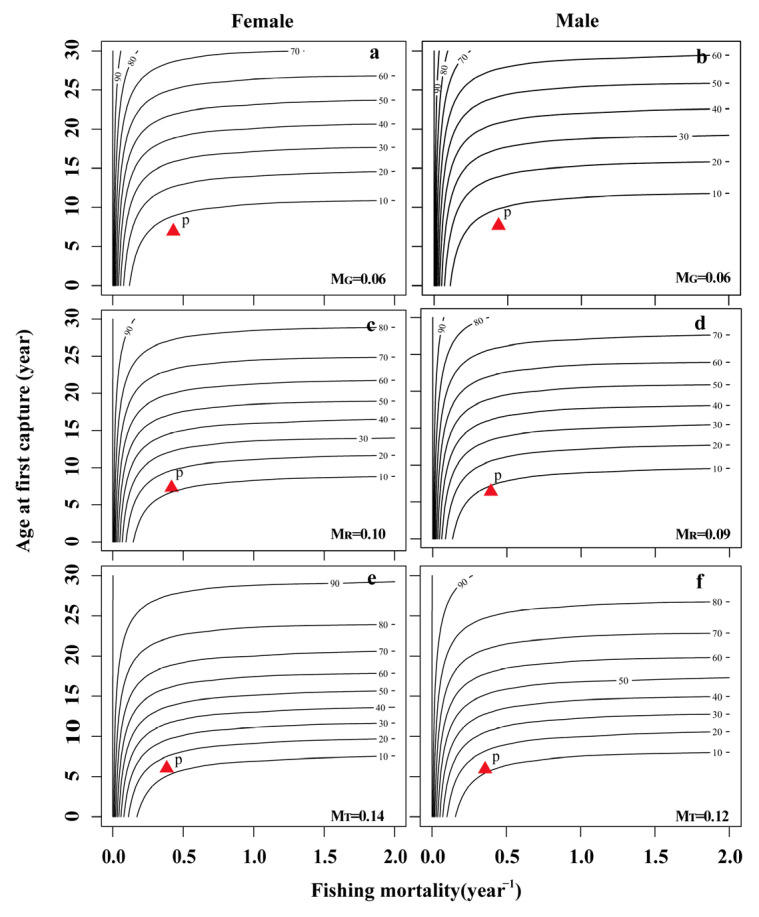
Isopleths of the spawning potential ratio (*SPR*) for *Schizothorax wangchiachii* in the upper reaches of the JSR. (**a**,**c**,**e**) The response of *SPR* to *t_c_* and *F* under different natural mortalities in female *S. wangchiachii*; (**b**,**d**,**f**) the response of *SPR* to *t_c_* and *F* under different natural mortalities in male *S. wangchiachii*. The “P” point represents the current estimated *SPR*. *M_T_*, natural mortality was calculated according to growth parameters; *M_G_*, natural mortality was estimated by the limit age method; *M_R_*, the reference natural mortality was calculated from the medians of *M_T_* and *M_G_*.

The catch per unit supplementary amount curve (X axis: fishing death coefficient F; Y axis: age at first capture (*t_c_*) was drawn (Figure 7 and Figure 8), and the optimal yield area in the figure was the area between Line A (the best t-point connection) and Line B (the best F-point connection). In the period from 2019 to 2020, the catch per unit supplementary amount corresponding to females (*F* = 0.36–0.44, *t_c_* = 5.49 a) and males (*F* = 0.41–0.47, *t_c_* = 5.36 a) in the current fishery area did not fall within the range of the optimal yield area, which implies that the utilization of the population resources of *S. wangchiachii* in the upper reaches of the JSR was in an overexploitation state (Figure 7 and Figure 8).

## 4. Discussion

The results of this study indicated that both the upper reaches of the JSR and the middle reaches of the YLR were in an overdeveloped state, which may be related to overfishing and population recovery ability. Due to overfishing and other factors, the catch of main fishing objects such as *S. wangchiachii* has decreased, with younger fish, most of which did not reach the age of sexual maturity, and the fish resources have shown a declining trend [10]. Phenomena such as excessive fishing and electric fishing were observed in the JSR Basin, leading to a sharp decrease in fish resources [43,44]. Overfishing led to a sharp decrease in the number of larger breeding individuals in the fish population, which would have a greater impact on the slow-growing and late sexual maturity schizothoracids [11,43]. The findings of this study showed that the growth parameter *k* value can be used to analyze life history strategies. The growth parameter *k* value depicted a slow-growing population at 0.05 to 0.10/year, an intermediately growing population at 0.10 to 0.20/year, and a fast-growing population at 0.20 to 0.50/year [45]. In this study, the growth parameter *k* value of the population in the middle reaches of the YLR was 0.0500, and the growth parameter *k* value of the population in the upper reaches of the JSR was 0.0608, indicating that the two populations of *S. wangchiachii* were slow-growing, and the resources were damaged and recovery would be difficult. Comparing the growth characteristics of different populations of schizothoracids (Table 4), the value of the growth parameter *k* of *S. wangchiachii* was less than 0.1, the asymptotic body length was greater, and the apparent growth index was between 4 and 5. The values of the parameters indicated that *S. wangchiachii* had slow growth and a long lifespan, and its growth characteristics conformed to the growth law of most schizothoracids.

The growth characteristic parameters of the same fish differed in various river sections [46,47]. In this study, by calculating the growth coefficient of the two populations, we concluded that the population of *S. wangchiachii* in the upper reaches of the JSR grows faster than that in the middle reaches of the YLR. There were many factors that contributed to these differences, including habitat differences among river sections (such as food abundance and water temperature), differences in the number and composition of samples, and differences in individual metabolic levels [47,48]. The larger the size of individuals obtained during sampling was, the larger their progressive body length and weight, the wider the range of body length or age, and the closer the growth characteristic parameters [47,49]. In addition, we also found a significant difference in the total mortality rate between male and female individuals in the YLR population, with females having a higher total mortality rate than males. However, the difference in total mortality rate between male and female individuals in the JSR population was relatively small, and females had a lower total mortality rate than males. In this study, compared with the YLR population, the JSR upstream population was represented by more samples, but its age range and size were smaller. This may be an important reason for the difference in growth parameters between the two populations.

In addition, fish are temperature-changing animals, and their feeding and growth will be affected by water temperature. Some scholars have noted that the different growth characteristics of different populations of schizothoracids were closely related to the environment; specifically, they were positively related to water temperature and food and negatively related to altitude and flow rate [50]. In this study, based on the growth coefficients of the two populations, we concluded that the population in the upper reaches of the JSR grows faster than the population in the middle reaches of the YLR. According to relevant research reports, the lowest annual average water temperature in the upper reaches of the JSR was 0.3 °C, with a short ice cover period. However, the middle reaches of the YLR had a long winter ice cover period, with a minimum water temperature of 0 °C [51]. In addition, the abundance of bait organisms in the middle reaches of the YLR was low [51,52]. In this study, the elevation of the sampling section in the middle reaches of the YLR was 2572 to 3230 m, the elevation of the sampling section in the upper reaches of the JSR was 1818 to 3408 m, and there was a significant relationship between river water temperature and altitude. This suggests that the regional differences in altitude and water temperature may be the main reasons for the growth differences between distinct geographic populations of *S. wangchiachii*. Similar results were also confirmed in a study of *Gymnodiptychus dybowskii* in different geographical groups of the Ili River [50].

To better develop fisheries management strategies, many scholars have advocated the use of biological reference points such as *F_Max_*, *F*_25%_ and *F*_40%_ [53,54,55]. The analysis results of *S. wangchiachi* in this study show that under the current three natural mortality rates, the *F_cur_* of the male and female populations was greater than *F*_25%_, which indicates that the population was overexploited. Thus, corresponding protection measures are needed. For example, the capture age of the female and male populations of *S. o’connori* increased to no less than 17 and 14 years old, respectively, ensuring that the reproductive potential ratio was always higher than *F*_25%_ [4]. However, there were differences in population parameters such as catch per unit recruitment, reproductive potential, and age of first sexual maturity between the JSR and YLR. If management was carried out according to the same population, the spatial heterogeneity of the population was ignored, which would lead to overfishing of fishery resources [56,57]. Therefore, the JSR and YLR groups should be treated as two protection units, and different protection measures should be formulated according to the resource status of each group.

Due to the current fishing intensity, the JSR population and the YLR population of *S. wangchiachii* were in an overexploitation state, the female starting age was lower than the age of first sexual maturity, and the male starting age was higher than the age of sexual maturity. The proportion of males and females and the number of breeding groups were bound to be threatened. Reducing fishing mortality and increasing fishing age could reduce fishing intensity and maintain high catches, which is a reasonable measure for resource management [1,4]. Combined with the variation trend of the spawning potential ratio and yield per recruit with the fishing intensity in this study, it was recommended to increase the starting age of the female and male populations of *S. wangchiachii* in the YLR to 15 and 11 years and the starting age of the female and male populations in the JSR to 12 and 13 years, respectively.

At the same time, the proportion of natural river sections in the main stream of the JSR was only 48.49%, mainly concentrated in the upper reaches of the JSR. The natural section of the middle reaches of the YLR accounted for only 25.37%, mainly distributed in the section from Yajiang County to the dam site of Yangfanggou Hydropower Station. Changes in the natural river environment had a negative impact on the completion of the life history of *S. wangchiachii* [1,51]. Therefore, we recommend strictly controlling the intensity of hydropower development, reasonably carrying out ecological dispatching, and ensuring sufficient ecological discharge; some inefficient and ecologically harmful small-scale hydropower infrastructure should be demolished to restore the natural habitat of the river, select representative tributaries to implement systematic ecological restoration, and restore river connectivity.

In addition, the biological and ecological information of *S. wangchiachii* is incomplete, which makes it impossible to accurately assess its population status and is not conducive to the development of related protection work such as artificial reproduction. Therefore, we suggest carrying out a comprehensive survey on the number of species, geographical distribution, population status and threat level, strengthen diversified scientific research, and provide more basic data for its protection [58,59].

Finally, given the comprehensive fishing ban in the Yangtze River Basin, it is necessary to strengthen the management of fishing bans in each survey area, establish a fishing ban management team for the mainstream and important tributaries, invest in more regulatory equipment, improve the regulatory reward and punishment system, and severely crack down on illegal fishing to ensure that the important waters of the mainstream and tributaries can be monitored in real time throughout the year [60].

## 5. Conclusions

Our study analyzed the growth characteristics and population dynamics of two different *S. wangchiachii* populations, including those from the upper reaches of the JSR and the middle reaches of the YLR. The initial sexual maturity age and the ages at first capture of the YLR population were higher than those of the JSR population, while the growth parameter *k* was lower than that of the JSR population. Under the current fishing intensity, the reproductive potential ratio of male and female fish in the upper reaches of the JSR and the middle reaches of the YLR was lower than the lower limit reference point *F*_25%_, and both populations were overfished. It is recommended to raise the age of arrest and implement arrest monitoring. Our study helps to reveal the growth characteristics and population dynamics of *S. wangchiachii* in the upper reaches of the Jinsha River and the middle reaches of the YLR and provides a basic reference for the formulation of biological protection measures.

## Figures and Tables

**Table 1:** The body length and weight of the two groups of *Schizothorax wangchiachii* in the Jinsha River Basin.

Groups	Sample Size			Body Length (mm)		Body Weight (g)	
	Female	Male	Total	Mean ± SE	Range	Mean ± SE	Range
YLR	77	84	187 (26)	229.1 ± 60.5 ^a^	101.0 to 401.0	232.6 ± 167.3 ^a^	20.6 to 1001.2
JSR	76	90	230 (64)	184.0 ± 44.2 ^b^	88.0 to 334.0	131.6 ± 99.15 ^b^	10.6 to 640.3

Note: SE, standard error; mm, millimeter; g, gram. Numbers in parentheses are the number of samples for which sex could not be determined. Different lowercase letters indicate a significant difference (*p* < 0.05).

**Table 2:** Comparison of three functions of two *Schizothorax wangchiachii* populations from the Jinsha River Basin.

	Models	$W=aL^{b}$	$L_{t}=L_{\infty }\left(1-e^{-k\left(t-t_{0}\right)}\right)$	$L_{t}=L_{\infty }\;e^{-e^{-k\left(t-t_{0}\right)}}$	$L_{t}=\frac{L_{\infty }}{1+e^{-k(t-t_{0})}}$
Groups					
YLR		$W=0.0131L^{3.0695}$	$L_{t}=70.07\left(1-e^{-0.05\left(t+0.5875\right)}\right)$	$L=45.61e^{-e-0.1675(t-5.036^{)}}$	$L=\frac{39.16}{1+e^{-0.298\;(t-6.12)}}$
JSR		$W=0.0231L^{2.9722}$	$L_{t}=61.48\left(1-e^{-0.0608\left(t+0.3762\right)}\right)$	$L=42.31e^{-e-0.1782\left(t-4.379\right)}$	$L=\frac{36.13}{1+e^{-0.3162\;(t-5.38)}}$
AIC(YLR/JSR)		—	66.49/80.10	77.42/91.05	75.72/81.82

Note: YLR, Yalong River; JSR, Jinsha River; *W*, body weight; *L,* body length; *L_t_,* total length at age *t* years; *L_∞_*, asymptotic total length; *t*_0_, theoretical age at zero total length; *a*, growth condition factor; *b*, allometric growth index; *k,* growth parameter; *AIC*, Akaike information criterion.

**Table 3:** Biological parameters for per-recruit analysis of *Schizothorax wangchiachii* in the Jinsha River Basin.

Parameters	YLR		JSR	
	Female	Male	Female	Male
*k*	0.0579 year^−1^	0.0683 year^−1^	0.0637 year^−1^	0.0559 year^−1^
*t*_0_	−0.3318 year^−1^	−0.3031 year^−1^	−0.2203 year^−1^	−0.7094 year^−1^
$L_{\infty }$	642.9 mm	594.3 mm	622.1 mm	574.1 mm
*t_c_*	6.82 year	7.10 year	5.49 year	5.36 year
*Z*	0.658 year^−1^	0.453 year^−1^	0.504 year^−1^	0.532 year^−1^
*M_G_*	0.06 year^−1^	0.07 year^−1^	0.06 year^−1^	0.06 year^−1^
*M_R_*	0.09 year^−1^	0.11 year^−1^	0.10 year^−1^	0.09 year^−1^
*M_T_*	0.13 year^−1^	0.15 year^−1^	0.14 year^−1^	0.12 year^−1^
*F_cur_*_(*MG*)_	0.59 year^−1^	0.38 year^−1^	0.44 year^−1^	0.47 year^−1^
*F_cur_*_(*MR*)_	0.56 year^−1^	0.34 year^−1^	0.40 year^−1^	0.44 year^−1^
*F_cur_*_(*MT*)_	0.52 year^−1^	0.30 year^−1^	0.36 year^−1^	0.41 year^−1^
*t_r_*	1 year	1 year	1 year	1 year
*a*	0.0127	0.0143	0.0343	0.0297
*b*	3.0744	3.0485	2.7800	2.8362
*T*_50_	7.09	5.41	6.09	5.19

Note: YLR, Yalong River; JSR, Jinsha River; *k*, growth parameter; *t*_0_, theoretical age at zero total length; *L_∞_*, asymptotic total length; *t_c_*, the ages at first capture; *Z*, total mortality; *M_T_*, natural mortality calculated according to growth parameters; *M_G_*, natural mortality estimated by the limit age method; *M_R_*, the reference natural mortality calculated from the medians of *M_T_* and *M_G_*; *F_cur_*, current fishing mortality; *t_r_*, age at recruitment; *a*, growth condition factor; *b*, allometric growth index; *T*_50_, ages at first sexual maturity.

**Table 4:** Estimates of fishing mortality (*F*) and YPR and SPR reference points for female and male *Schizothorax wangchiachii* in the upper Jinsha River and in the middle reaches of the Yalong River.

Group	*M* (year^−1^)	*F_cur_* (year^−1^)	*F*_25%_ (year^−1^)	*F*_40%_ (year^−1^)	*F_max_* (year^−1^)	*YPR_cur_* (g)	*YPR*_25%_ (g)	*YPR*_40%_ (g)	*YPR_max_* (g)	*SPR_cur_* (%)
YLR	Female									
	0.06	0.59	0.09	0.05	0.07	288.47	198.25	156.13	290.12	2.92
	0.09	0.56	0.12	0.07	0.10	168.92	140.97	117.90	1+ 69.13	6.66
	0.13	0.52	0.17	0.09	0.15	165.85	91.73	82.02	96.39	11.25
	Male									
	0.07	0.38	0.10	0.06	0.09	125.30	157.44	121.37	207.80	5.91
	0.11	0.34	0.14	0.08	0.13	133.86	98.85	81.43	110.63	11.24
	0.15	0.30	0.21	0.11	0.17	146.13	63.52	55.23	65.93	18.57
JSR	Female									
	0.06	0.44	0.06	0.04	0.06	107.85	234.85	221.95	234.85	2.38
	0.10	0.40	0.09	0.06	0.09	87.88	134.82	128.99	134.82	4.94
	0.14	0.36	0.12	0.07	0.12	76.75	99.62	94.20	99.62	7.59
	Male									
	0.06	0.47	0.08	0.05	0.07	85.11	193.21	189.74	197.76	1.85
	0.09	0.44	0.11	0.06	0.10	64.47	100.26	93.48	100.38	3.91
	0.12	0.41	0.13	0.08	0.14	53.66	67.98	63.74	67.99	6.50

Note: YLR, Yalong River; JSR, Jinsha River; *M*, natural mortality rate, the three values of M represent from top to bottom: *M_T_*, natural mortality calculated according to growth parameters; *M_G_*, natural mortality estimated by the limit age method; *M_R_*, the reference natural mortality calculated from the medians of *M_T_* and *M_G_*; *F_cur_*, current fishing mortality; *F_max_*, fishing mortality rate at the maximum *YPR*; *F*_25%_ and *F*_40%_, fishing mortality rates at which the *SPR* is 25% and 40%; *YPR_cur_*, current yield per recruit; *SPR_cur_*, current spawning potential ratio; *YPR*_25%_ and *YPR*_40%_, yield per recruit at which the *SPR* is 25% and 40%; *YPR_max_,* the maximum yield per recruit.

## Data Availability

The data described in this study can be obtained from the corresponding authors. The data set is not publicly available because of its size.
